# Case management training needs to support vocational rehabilitation for case managers and general practitioners: a survey study

**DOI:** 10.1186/1472-6920-14-95

**Published:** 2014-05-16

**Authors:** Evangelia Demou, Mairi Gaffney, Furzana Khan, John K Lando, Ewan B Macdonald

**Affiliations:** 1 Institute of Health and Wellbeing, College of Medical, Veterinary and Life Sciences, University of Glasgow, Glasgow, UK; 2 Motherwell Health Centre, 138 Windmillhill Street, Motherwell ML1 1 TB, UK

**Keywords:** Case management, Condition management, General practitioners, Vocational rehabilitation, Training needs

## Abstract

**Background:**

The use of the biopsychosocial model of health and case management for effective vocational rehabilitation (VR) has been confirmed for many health conditions. While Case and Condition Managers (CCMPs) use this approach in their everyday work, little is known about their views on training needs. A review of the training curriculum for General Practitioners’ (GPs) revealed little training in VR and the biopsychosocial model of care. This study aims to identify Case and Condition Managers and GPs perceptions of their training needs in relation to employability and VR.

**Methods:**

80 Case and Condition Managers and 304 GPs working in NHS Lanarkshire, providing a comparison group, were invited to participate in this study. A self-completion questionnaire was developed and circulated for online completion with a second round of hardcopy questionnaires distributed.

**Results:**

In total 45 responses were obtained from CCMPs, 5 from occupational health nurses (62% response rate) and 60 from GPs (20% response rate). CCMPs and the nursing group expressed a need for training but to a lesser extent than GP’s. The GP responses demonstrated a need for high levels of training in case/condition management, the biopsychosocial model, legal and ethical issues associated with employment and VR, and management training.

**Conclusions:**

This survey confirms a need for further training of CCMPs and that respondent GPs in one health board are not fully equipped to deal with patients employability and vocational needs. GPs also reported a lack of understanding about the role of Case and Condition managers. Training for these professional groups and others involved in multidisciplinary VR could improve competencies and mutual understanding among those advising patients on return-to-work.

## Background

Vocational Rehabilitation (VR) encompasses assistance to a broad group of people, including individuals with physical and mental-health problems. The VR Scottish Executive Framework defines it as: *“a process that enables people with functional, psychological, developmental, cognitive and emotional impairments or health conditions to overcome barriers to accessing, maintaining or returning to employment or other useful occupation”*[1]. Its focus is to help people retain or regain the ability to work, rather than to treat illness or injury [2]. VR can require input from different professional disciplines, including clinicians, disability advisers and career counsellors, and the techniques used can include assessment and appraisal, goal setting, intervention planning, and provision of health advice and promotion [3,4].

Vocational rehabilitation is central to the health needs of the working-age population, but there is a perceived lack of both VR facilities and trained individuals within health provision to deliver it [5]. Dame Carol Black’s report suggested that this would require the creation of a workforce of professionals equipped with what are primarily non-medical multidisciplinary skills, such as case management skills, and the ability to undertake a holistic assessment of patient/client needs [6]. Although medical problems might be the basis of such a plan, additional approaches could include cognitive behavioural therapy (CBT) and, support and advice for social problems. Dame Black’s report elucidated the underlying principle of such an approach as one of ‘holistic care’ including the ‘biopsychosocial model’ [6]. The latter simultaneously considers the biological, psychological and social determinants that may negatively impact on health and well-being including work, as well as the links between all three factors [7]. The Peninsula Medical School reported that an array of medical issues, including musculoskeletal, cardiorespiratory, and mental-health problems, could be treated using a biopsychosocial model [8]. The importance of early VR is widely agreed, and ideally should be part of every health-care treatment plan [9], even before a person starts receiving benefits [10]. A number of approaches have been identified for managing the rehabilitation process: self-management, condition and case management [1]. Case Management Society UK define Case Management as: *“a collaborative process which: assesses, plans, implements, co-ordinates, monitors and evaluates the options and services required to meet an individual’s health, social care, educational and employment needs, using communication and available resources to promote quality cost effective outcomes”*[11]. Condition Managers were employed specifically for the DWP Pathways to Work programme and had a similar role to Case Managers but with a lesser ability to arrange treatments for individuals [12,13]. The important role of case management in vocational rehabilitation is highlighted in the literature [14-18]. Common barriers to rehabilitation include the priority given to other medical conditions, economic factors, coupled with a lack of top-level organisational commitment and a coordinated approach among rehabilitation providers [19].

Family doctors and primary-care teams are the patient’s initial source of advice about fitness-to-work, but they generally lack adequate training or expertise in occupational health or disability evaluation and often do not understand or consider occupational issues or the consequences of long-term incapacity [5,20]. Specific gaps identified are in health professionals understanding of the relationship between health and work and of alternatives to or options to minimise sickness [5]. Farrell et al. [21] offered similar findings, reporting that for issues relating to return-to-work, participants seemed not to ask GPs for advice because GP advice often did not concur with participants’ own views. Rehabilitation providers who had been encouraging participants to consider return-to-work felt that GPs sometimes advised against it [21]. Frank and Chamberlain [22] found that rehabilitation-service providers were dissatisfied with GPs who provided long-term sick notes, which they felt could deflate clients confidence and discourage returning to work. Examination of the Royal College of General Practitioners’ curriculum, to assess current GP training in relation to VR and the biopsychosocial model, found that while the curriculum mentioned VR and the biopsychosocial model, these were in relation to coordinating with non-medical agencies and that GP training did not specifically focus on aspects of core training that might be appropriate for working with other professionals on employment issues related to VR and the biopsychosocial model [23].

The aim of this study was to identify Case and Condition Managers and GPs perceptions of their training needs in relation to employability and vocational rehabilitation requirements of their clients and patients, using a self-completion questionnaire.

## Methods

Competency questions were identified to include the different dimensions of VR. A self-completion questionnaire was then developed and piloted among Case/Condition Managers (CCMPs) in Lanarkshire (n = 8). Only small editorial changes resulted from this pilot study. Questions were then amended as required prior to being posted as a web-based survey. Survey options in the self-administrated questionnaire were ‘No; Low; Medium; or High Training Need’.

One researcher attended a meeting with the Director of CMP (Glasgow City Regional Lead, CMP, Scotland) to encourage participation from CCMPs in Scotland and Manchester. All Case and Condition Management Practitioners (CCMPs), a group primarily involved in vocational rehabilitation, throughout Scotland and one group from Manchester were invited to participate in the online survey. This provided the opportunity of a comparative group to the GPs of potentially more ‘informed’ practitioners on the specific topic.

Participation by GPs was much lower. Members of the research team attended meetings with ‘key contact’ GPs inviting them to participate in the survey. A list of potential participants for the survey was compiled and emails were sent out on two occasions. Personal visits to individual GPs were also made. All GP practice managers within the Lanarkshire area were identified through an Internet search and a total of 100 were contacted seeking their advice on how to encourage GP participation and offering a range of options including online survey completion, postal survey, telephone interview, or face-to-face interviews. Most practice managers were willing to assist in bringing the survey to the attention of their GPs, but some felt that GPs simply would not participate because they either had a policy not to, or that the timing of the survey was incompatible with GP workload. Practice managers advised that GPs would be most likely to complete postal (92%) and email surveys (8%), whereas telephone and face-to-face interviews would not be favourable.

The Welsch t-test was performed to determine whether there is a statistically significant difference between the mean response of GPs and CCMPs with regards to their training needs in vocational rehabilitation issues.

Ethical approval was sought from the NHS Lanarkshire Ethical Committee which advised that it was not required for this investigation.

## Results

A total of 304 GPs were ‘encouraged’ by their practice managers to participate upon receipt of the postal and email survey and after follow-up calls by the researcher. By the end of the allocated field work period a total of 53 postal surveys and 7 online surveys were returned. No further responses to the web-based survey occurred (some blank surveys were returned with apologies for ‘no interest’ in the survey). In total 60 GPs responded.

There was a relatively speedy participation among the 80 potential CCMP respondents (full and part-time workers). In total 45 CCMPs participated in the online survey, and a small group (n = 5) of Occupational Health related professionals (nurses) responded, utilising the online survey.

As the Case and Condition Managers serve as a comparative group of potentially more ‘informed’ practitioners on the specific topic, the results of the vocational rehabilitation needs of GPs are presented first and the results for the training needs of CCMPs are presented with respect to the GP results.

### Training needs of general practitioners

The results demonstrated four categories for which more than 50% of the GPs required ‘Medium’ and ‘High’ training needs (Table 1). These were the categories of ‘Training needs related to Case/Condition Management’ , ‘The Biopsychosocial Model’, ‘Legal & Ethical Issues associated with Employment and VR’, and ‘Management Training’. In terms of the training needs related to Case/Condition Management, the GPs noted the highest training needs for the sub-categories of ‘Developing a return-to-work plan with stakeholders’ (74%) and ‘Assessing the return-to-work needs of their patients’ (72%). The overall average of medium and high training needs of this category was 65%.

**Table 1:** GP and case and condition manager training needs

**Training needs**	**GP (%)**	**CCMP (%)**
	**[N = 60]**	**[N = 45]**
***Training needs related to Case/Condition Management***		
Development of a return to work plan with stakeholders	74^*^	57
Assessing the return to work needs client/patients	72	52
Understanding the principals and practice of Vocational Rehabilitation	65	58^*^
Coordinating a range of support within, and out with organisations to allow return to work, and/or facilitating the retention of the client/patient in their work place	**65**	**55**
Assessing workplace factors that impact on outcomes of client/patient return to work	63	57
Demonstrating functional knowledge of ergonomics	**63**	**51**
Development of skills and understanding of case management	**53**	**50**
***The Biopsychosocial Model***		
Understand how to move the ‘locus of control’ back to the client/patient	71^*^	49
Understanding the Concept of ‘Workability’	67	42
Knowing how to reduce unnecessary sickness absence	**60**	**43**
Identify those in work for early intervention	55	43
Understanding the principals of the Bio-Psycho- Social Model	46	36
Identify those not in work for early intervention	45	51^*^
Understanding Psycho-Social aspects that affect client/patient health care	44	37
Understanding positive health and social aspects of ‘good work’	35	43
***Legal & Ethical Issues Associated With Employment and Vocational Rehabilitation***		
Disability Discrimination Act	73^*^	54
Employment Rights	73^*^	60
How codes of conduct effect delivery of employment of VR	65	61
Knowledge of the basic requirements of Health & Safety Law	57	49
Equal opportunities Legislation	55	62^*^
***Common Conditions Associated With Employment and Vocational Rehabilitation***		
Mental Health Conditions and Treatment	43^*^	40
Musculoskeletal disorders and Treatment	**40**	**58**
Chronic Degenerative Conditions and Treatment	35	62^*^
***Assessment and Measurement Tools***		
Interpret data for the Purpose of Action Plan	40^*^	46
Assessment Tools Relevant to Case Management	35	47^*^
***Management Training***		
Operational Management Skill	62^*^	64^*^
Leadership Skills	**50**	**58**
Appreciating the Priorities of those who Manage Organisations	**50**	**53**
***Awareness of the Roles of Professionals Working in Relation to Employment VR***		
Knowledge of Case Manager role	80^*^	20
Knowledge of Condition Management role	64	20
Knowledge of Employment Advisor role	57	18
Knowledge of Occupational Therapist role	38	13
Knowledge of General Medical Practitioner role	36	11
Knowledge of Community Nurse (District) role	36	33
Knowledge of Public Health (Health Visitor) Nurse role	34	39^*^
Knowledge of Practise Nurse role	30	34
Knowledge of Occupational Health Nurse role	30	18
Knowledge of Occupational Health Physician role	27	35
Knowledge of Physiotherapist role	**25**	**11**

Percentage of respondents reporting medium and high training needs.

Bold and superscript * indicates the subcategory with the highest training need.

Bold indicates the subcategories for which the training needs of GPs and CCMPs were ranked in the same position.

The answers related to GPs awareness of the roles of professionals working in relation to employment VR, were in line with the above findings. Only three categories demonstrated medium and high training needs of more than 50%, and these were on the roles of Case and Condition Managers (80% and 64%, respectively) and Employment advisers (57%). In terms of the biopsychosocial model, GPs training needs appear to be split in two. For half of the subcategories more than 50% expressed medium to high training needs and less than 50% for the other half. Their understanding of the biopsychosocial model, psycho-social aspects that affect health and positive aspects of ‘good work’ appears better than issues of transferring control back to the patient (71%), workability (67%), and sickness absence (60%). Training was required more for the identification of those individuals who were in work and were in need of an early intervention (55%) to prevent them from going off work, than those who were not in work (45%).

A number of topics were raised from the written comments provided by the GPs and these included:

• The questions asked were not related to GPs

• Some commented that GPs should either not be or are already too involved in return-to-work assessment

• Some felt that training in the areas and issues raised was welcomed and expressed that this is a training area some may already be involved in

• Training was required in understanding and filling in forms within legal and clinical competence, especially when they do not know details of a patient’s job.

### Training needs of case and condition managers

Of the 80 Case/Condition Managers surveyed, 56% responded. Of the 45 who responded, 5 were Case Managers and 40 were Condition Managers, mostly (68%) from the areas of Lanarkshire, Glasgow, and Ayrshire and Arran.

Similar to the GPs for the category of ‘Training needs related to Case/Condition Management’ more that 50% of the CCMPs reported medium and high training needs (Table 1). CCMPs training needs did not appear as high as for GPs (average over all subcategories 54%) and the subcategory ranking highest in terms of training needs was ‘Understanding the principals and practice of VR’. For three subcategories GPs and CCMPs training needs ranked the same (Table 1). The second category for which more than 50% of CCMPs reported medium and high training needs was that of ‘Management Training’, for which the subcategories displayed the same ranking as for GPs.

Relatively high training needs for CCMPs were expressed for the categories of ‘Legal & Ethical Issues associated with Employment and VR’ and ‘Common Conditions associated with Employment and VR’. For all but one subcategory in these, more than 50% of CCMPs reported medium and high training needs. In both cases the highest training need for CCMPs was the lowest for GPs (Table 1). CCMPs appeared better trained in the biopsychosocial model, with only one category receiving more than 50% (Table 1).

Overall, there was no statistical difference found in training needs between GPs and CCMPs except in relation to ‘Training Needs Related to Case/Condition Management’ (p < 0.01) and ‘Awareness of the Roles of Professionals Working in Relation to Employment Vocational Rehabilitation’ (p < 0.01). For both of the above categories a greater percentage of GPs reported medium and high training needs (Table 1).

Themes that emerged from the comments of CCMPs included:

• Some had taken specific training at MSc level in VR and noted that VR knowledge in the NHS was limited;

• There were standard training courses that CCMPs undertook, but that continual training was essential in their job;

• Standardisation need across CCMPs nationally with respect to models and practices, and solid evaluation of health and vocational outcomes for clients who receive input from CCMPs;

• The level of support received in the workplace for their skills/development improvements was adequate;

• Seven areas of training were emphasized: motivational interviewing and health behaviour change; functional capacity evaluations within the workplace; health and work focused goal planning; partnerships between health and work agencies; different models of intervention; solution focused therapy; needs related training.

### Occupational health-related professionals

The five occupational health related professionals (nurses) came from four health boards. However, due to the small sample size no representative conclusions can be formally drawn. However, the results are presented as they may be indicative as to a possible ‘need’ for further research into the training needs of this group. Similar to the other two professional groups, the highest demand for training was reported for the ‘Training needs related to Case/Condition Management’. Only one occupational health related practitioner expressed a need for further training on different intervention models.

## Discussion

In order to establish what training needs there might be among Case and Condition Managers and General Practitioners a survey was conducted and the two groups were compared. The findings of this study show that two years after the publication of the Black report [6] and in line with the findings of Waddell and Burton [5] that general practitioners require further training to improve their ability to manage the VR of their patients and provide competent advice about their fitness-for-work, there was a strong need for training, not just among respondent GPs, but also to a slightly lesser extent among Case/Condition Managers. The need for training was found to be largely in all areas that are associated with: case/condition management; the biopsychosocial model; legal & ethical issues associated with employment and VR; and management training. Specific areas where GPs were significantly more likely to require training were highlighted, such as in relation to the roles of Case/Condition Managers and Employment advisors. Areas that few GPs (30% or less) were found to require training in were: Knowledge of Practice Nurse Role; Knowledge of Occupational Health and Physician Role, and Knowledge of Physiotherapist Role.

Three GPs expressed no interest in involvement or too much involvement already in VR. However, recent research has tried to fill the gap of knowledge of case/condition management and the biopsychosocial model in primary care [10,24-27]. Gensichen et al. [24] reported that the implementation of case management in their practice was not only beneficial for the patient, but that it enhanced their consultation styles and their relationships with their patients and their practice team. Wermeling et al. [27] suggested that some GPs addressed psychological issues more often when patients repeatedly presented with chronic neck pain and that GPs training and presence or absence of an additional qualification in psychosomatic medicine significantly affected this [28].

One GP addressed limitations to their work, using the example of *“patients job/truth of issues not necessarily known”.* Such hindrances and others to their work have been reported. For instance, GPs report difficulties in addressing psychosomatic factors as they feel that many patients cannot accept a psychological explanation and demand therapies for their ‘real’ pain and cannot establish a connection between somatic symptoms and psychological influences [27]. In many cases, the prescription and referral does not necessarily reflect a lack of knowledge but the GPs’ strategic decision to improve the doctor-patient relationship [27].

This survey revealed that the perceived training need of CCMPs was relatively high. This was unexpected given that this professional group worked in VR, routinely used the principles of the biopsychosocial model and had training in these areas. They also represent a relatively expert group, working directly with long-term workless clients with chronic health problems, with the aim of assisting them in a ‘holistic’ way to return to or undertake employment. Especially for the category of ‘Training related to Case/Condition Management’ more than 50% of CCMPs reported medium and high training needs. Further training of CCMP’s in these areas is recommended as part of their continuing professional development.

The need for improved collaboration between different professional groups has been recognised with the aim of promoting a more holistic approach at improving health care, job retention and return-to-work following sickness [28-32]. The survey results suggest the current training does not amply equip GP’s with the competencies, skills and knowledge to adequately deal with employability, VR, and fitness-to-work. Also their training does not appear to fully cover the practice of working with professionals outside the clinical sphere. If GPs are to be able to deal with patient employability and VR needs, changes may have to be made to their core training. The lack of awareness of other disciplines and agencies involved in this area could be addressed by more integrated case-based training to equip GPs with knowledge of the other professions and resources in multidisciplinary VR. This would improve their knowledge of the resources available both within and without the NHS. The survey data showing similar training ‘needs’ amongst GPs and CCMPs, indicates that joint training of these groups may provide a common understanding and enhance collaborative working. A common language and understanding would enable them to take advantage of each other’s areas of expertise and consequently enhance the services they are providing.

Previous studies have shown that Case and Condition Management programmes (CMPs) within primary care can be beneficial and provide a mechanism to facilitate return-to-work for individuals with health problems [10,24-27]. Joyce et al. [10] highlighted the fact that although such programmes tend to be delivered by allied health professionals, GPs could support them and their patients during the return-to-work process. Stable relations with the healthcare provider, as well as the establishment of questions to patients on their perceptions and expectations of returning to work could be beneficial in the management of illness and reducing sickness absence [33,34]. Such a cross-cutting, multiagency and multidisciplinary approach before, during and after job loss could be beneficial to patients [10,26,34,35].

Despite numerous efforts only a low response rate of 20% was achieved from the GPs. Low response rates to postal and online surveys are repeatedly reported in the literature for general practitioners [36-41]. This issue raises concerns for the presence of response bias. A number of studies investigating non-responders within the GP population have identified that they tend to be older, have a low interest in the topic, are single-practise GPs, and are less qualified than responders [36,37,40-42]. While this brings into question the generalizability of the results of this survey, previous studies have demonstrated that there is no threshold of acceptable response rates and that even high response rates do not escape non-response bias [36,41-43]. Additionally, we were not able to compare the responders with the study population, as detailed demographic details were not collected to ensure anonymity of the participants. While this may be a limitation due to the low response rate, it does not invalidate the findings of this survey, as Barclay et al. [44] for instance, found significant differences in all demographic variables between responders and non-responders even though they reported a 86.7% response rate.

## Conclusions

This survey confirms a need for further training of CCMPs, which was unexpected given that this professional group works in VR, routinely uses the principles of the biopsychosocial model and receives training in these areas. The low response rate from the GPs prevents from generalising the results to the entire GP population. However, the findings from the respondent GPs in the health board assessed, show that they are not fully equipped to deal with patients’ employability and vocational needs. GPs also reported a lack of understanding about the role of Case and Condition managers. Training for these professional groups and others involved in multidisciplinary VR could improve competencies and mutual understanding among those advising patients on return-to-work.

## Competing interests

The authors declare that they have no competing interests.

## Authors’ contributions

ED was the main author, analysed the data and interpreted the results. MG undertook the literature review for the competency questions on vocational rehabilitation, assisted in developing and piloting the questionnaire. FK was the main researcher who conducted the survey, gathered the data and assisted in the analysis. JKL was the advisor on accessing General Practitioners, facilitated GP recruitment and advised about the design of the study. EBM originated the proposal for the study, coordinated its execution and co-authored the manuscript. All authors read and approved the final manuscript.

## Pre-publication history

The pre-publication history for this paper can be accessed here:

http://www.biomedcentral.com/1472-6920/14/95/prepub
